# PW01-004 – The sequence analysis in E148Q homozygous patients

**DOI:** 10.1186/1546-0096-11-S1-A57

**Published:** 2013-11-08

**Authors:** R Topaloglu, C Yildiz, E Taskiran, E Korkmaz, N Besbas, S Ozen, A Duzova, N Akarsu, F Ozaltin

**Affiliations:** 1 Pediatrics, Hacettepe University, Ankara, Turkey; 2 Medical Genetics, Hacettepe University, Ankara, Turkey; 3 Nephrogenetic lab, Hacettepe University, Ankara, Turkey

## Introduction

Familial Mediterranean fever (FMF) is an autosomal recessive disease associated with a number of mutations of the MEFV gene. To date 246 variants responsible for the disease were identified, one such a variant is E148Q in exon 2. The role of E148Q variant in the development of FMF remains inconclusive. Some authors believe it causes the disease, whereas others favor the concept of a non causative role.

## Objectives

The aim of this study was to perform MEFV gene sequence analysis in E148Q homozygous patients in order to detect an associated novel mutation/variant; to determine the frequency of E148Q allele in healthy society, and ultimately, to clarify the controversy about whether E148Q is a disease causing mutation or a benign polymorphism.

## Methods

Patients with homozygous E148Q variation previously determined by strip assay were evaluated. FMF clinical forms were filled in for those. All coding exons including exon-intron boundaries of MEFV gene were sequenced using DNA obtained from peripheral blood lymphocytes. E148Q allele frequencies of the parents and that of control group consisting of 100 healthy individuals were determined.

## Results

Eighteen E148Q homozygous patients were studied. Age at the onset of disease was 5±3.7 (median 5) years. 44% of the patients were males and 56% were females. Presenting manifestations were abdominal pain (78.5%), fever (71.4%), arthralgia (57.1%), pleuritis (28.5%) and myalgia (7.1%). With colchicine treatment, significant decrement in annual number of attacks, duration of attacks and disease activation scores was observed (p<0.05). There was no difference in terms of achievement of full or partial remission, possessing low or moderate disease activation score, living without attack or with >20 attacks/year between groups carrying associated variants (detected as R314R, P369S, R408Q, E474E, Q476Q, D510D, P588P) and those with no associated variants. In control group consisting of 100 healthy individuals, the frequency of E148Q allele was found to be 6.5%.

## Conclusion

The frequency of E148Q variant in healthy population was found to be 6.5% in contrast to a previous report as 12% in Turkey. The E148Q variant may be related with disease since most of the patients carrying this specific variant were symptomatic (78% of our patients) and responsive to colchicine treatment, and no other variants in any coding region of the gene were detected by direct sequence analysis to be responsible for the phenotype.

## Disclosure of interest

None declared

